# Supplementary material for: Complexome profiling of the Chlamydomonas psb28 mutant reveals TEF5 as an early PSII assembly factor
Source: Plant Cell. 2025 Mar 17;37(6):koaf055. doi: 10.1093/plcell/koaf055 (PMC12164590; doi:10.1093/plcell/koaf055)
Supplement: koaf055_Supplementary_Data [file koaf055_supplementary_data.zip › psb28_interactive.zip!/plots/intensity/qualityPlots/peptides/CURTL2_Cre10.g433950.t1.1.html]

### Cre10.g433950.t1.1

Trivial name: CURTL2  
SeqCoverage [median]: 37.10  
Mass: 16.09 kDa  
  
 

| Sequence | MissedCleavage | Mass [Da] | wtIntensity | muIntensity | Score |
| --- | --- | --- | --- | --- | --- |
| AEPESSTSAEDPSVK | 0 | 1532.68 | 53015.01 | 87658.43 | 225.19 |
| AEPESSTSAEDPSVKLQK | 1 | 1901.92 | 69703.09 | 41239.76 | 110.37 |
| KSIDAFVK | 1 | 906.52 | 0.00 | 373.89 | 66.69 |
| LQKQAEEVLK | 1 | 1184.68 | 1448.14 | 4451.78 | 152.54 |
| QAEEVLK | 0 | 815.44 | 145579.69 | 158569.69 | 172.03 |
| QAEEVLKQVQGK | 1 | 1355.74 | 26700.43 | 28719.93 | 167.18 |
| SIDAFVK | 0 | 778.42 | 196451.85 | 106165.56 | 153.15 |
| SIDAFVKSVTGEK | 1 | 1379.73 | 0.00 | 14180.18 | 68.03 |
| YFTDPAER | 0 | 997.45 | 15712.23 | 41551.54 | 168.45 |
| YFTDPAERESVK | 1 | 1440.69 | 0.00 | 518.81 | 36.98 |
| YFTDPAERESVKK | 2 | 1568.78 | 4162.85 | 6640.32 | 78.90 |
|  |  |  | 512773.3 | 490069.9 |  |
